# The γ-gliadin multigene family in common wheat (*Triticum aestivum*) and its closely related species

**DOI:** 10.1186/1471-2164-10-168

**Published:** 2009-04-21

**Authors:** Peng-Fei Qi, Yu-Ming Wei, Thérèse Ouellet, Qing Chen, Xin Tan, You-Liang Zheng

**Affiliations:** 1 Triticeae Research Institute, Sichuan Agricultural University, Yaan, Sichuan 625014, PR China; 2 Ministry of Education Key Laboratory for Crop Genetic Resources and Improvement in Southwest China, Sichuan Agricultural University, Yaan, Sichuan 625014, PR China; 3 Agriculture & Agri-Food Canada, Eastern Cereal and Oilseed Research Centre, Ottawa, Ontario, K1A 0C6, Canada

## Abstract

**Background:**

The unique properties of wheat flour primarily depend on gluten, which is the most important source of protein for human being. γ-Gliadins have been considered to be the most ancient of the wheat gluten family. The complex family structure of γ-gliadins complicates the determination of their function. Moreover, γ-gliadins contain several sets of celiac disease epitopes. However, no systematic research has been conducted yet.

**Results:**

A total of 170 γ-gliadin genes were isolated from common wheat and its closely related species, among which 138 sequences are putatively functional. The ORF lengths of these sequences range from 678 to 1089 bp, and the repetitive region is mainly responsible for the size heterogeneity of γ-gliadins. The repeat motif **P**(Q/L/S/T/I/V/R/A)**F**(S/Y/V/Q/I/C/L)**P**(R/L/S/T/H/C/Y)**Q**_1–2_**(P**(S/L/T/A/F/H)**QQ)**_1–2_is repeated from 7 to 22 times. Sequence polymorphism and linkage disequilibrium analyses show that γ-gliadins are highly diverse. Phylogenic analyses indicate that there is no obvious discrimination between *Sitopsis *and *Ae. tauschii *at the *Gli-1 *loci, compared with diploid wheat. According to the number and placement of cysteine residues, we defined nine cysteine patterns and 17 subgroups. Alternatively, we classified γ-gliadins into two types based on the length of repetitive domain. Amino acid composition analyses indicate that there is a wide range of essential amino acids in γ-gliadins, and those γ-gliadins from subgroup SG-10 and SG-12 and γ-gliadins with a short repetitive domain are more nutritional. A screening of toxic epitopes shows that γ-gliadins with a pattern of C9 and γ-gliadins with a short repetitive domain almost lack any epitopes.

**Conclusion:**

γ-Gliadin sequences in wheat and closely related *Aegilops *species are diverse. Each group/subgroup contributes differently to nutritional quality and epitope content. It is suggested that the genes with a short repetitive domain are more nutritional and valuable. Therefore, it is possible to breed wheat varieties, the γ-gliadins of which are less, even non-toxic and more nutritional.

## Background

The unique properties of wheat flour primarily depend on seed storage proteins, which mainly consist of gluten [1]. Gluten is glutamine and proline-rich proteins, having a storage function of nitrogen and sulphur. Gluten is traditionally classified into low molecular weight (LMW) glutenins, high molecular weight (HMW) glutenins and the large gliadin group [2]. Gliadins are mainly monomeric proteins of 30–78 kD with poor solubility in dilute salt solutions, and good solubility in 70% ethanol [3]. The gliadins are composed of α-, γ- and ω-types [4]. HMW-glutenin genes locate at the long arms of group 1 chromosomes (*Glu-1 *loci) [5]. The α-gliadins are encoded by the *Gli-2 *loci on the short arms of group 6 chromosomes. The γ-gliadins and ω-gliadins are encoded by the *Gli-1 *loci on the short arms of homeologous chromosome 1, and are tightly linked to the *Glu-3 *loci coding for LMW-glutenins [6-8].

Gluten is the most important source of protein for human being, and a wide diversity of food has been developed to take advantage of the properties (i.e. mixing characteristics, dough rheology and baking performance) of wheat flour. It is reported that visco-elastic properties of wheat are affected by the proportions of gluten polymers, and that allelic variation in the composition of the HMW-glutenins is strongly correlated with differences in the breadmaking quality [2,9]. Although gliadins comprise of 40–50% of total endosperm storage proteins in wheat, their roles in determining the properties of wheat flour are not well understood yet. The complex family structure of gliadins, compared to HMW-glutenin, complicates the determination of their function. The estimated copy number for γ-gliadin genes is between 15 and 40 in *Triticum aestivum *cv 'Chinese Spring' [10]. Classification based on their primary structure would facilitate further functional studies. γ-Gliadin genes have previously been divided into three hybridization classes [11]. Pistón et al. [12] classified γ-gliadin genes into four groups based on the phylogenic analysis.

Celiac disease (CD), a widely prevalent autoimmune disease of the small intestine (above 1:200 in most population groups), is induced in susceptible individuals by exposure to dietary gluten [13]. However, given the enormous biological diversity and unique chemistry of gluten, and the absence of satisfactory assays for gluten toxicity, the structural basis for gluten toxicity in CD remains unclear [14]. It has been shown that some native gluten sequences can bind to HLA-DQ2/8 and induce T cell responses. In addition, modification of gluten peptides by the enzyme transglutaminase results in high affinity HLA-DQ2/8 binding peptides that can induce T cell responses [15,16]. The principal toxic components of wheat gluten are gliadins. γ-Gliadins contain several sets of celiac disease epitopes [17].

The γ-gliadins have been considered to be the most ancient members of the wheat gluten family [18]. Sequence information for γ-gliadin genes in GenBank includes 34 complete/nearly complete open reading frame (ORF) and 66 partial sequences. These sequences come from various wheat and *Aegilops *species. However, no systematic research has been conducted yet.

We have performed an extensive and comparative analysis of γ-gliadin genes from common wheat and its closely related species in order to classify the γ-gliadin genes, and to investigate the diversity of the CD epitopes and nutritional quality of each γ-gliadin group. This study clarifies our understanding of the evolution of the multigene family.

## Results

**Figure 1:**
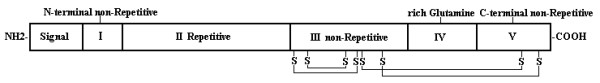
**Model structure of γ-gliadins**. The amino acid sequence starts with a 20-residue signal peptide, followed by a short N-terminal non-repetitive domain (I), a highly variable repetitive domain (II), a non-repetitive domain containing most of the cysteine residues (III), a glutamine-rich region (IV), and the C-terminal non-repetitive domain containing the final two conserved cysteine residues (V). All the eight conserved cysteine residues form intramolecular disulphide bonds.

A total of 170 γ-gliadin genes were isolated from common wheat and its closely related species (Table 1). There is no indication of introns interrupting the coding region (Figure 1). Thirty-two of these sequences are pseudogenes, all of which contain one or more internal stop codons or frameshift mutations caused by single nucleotide indels (insertions/deletions). The remaining 138 sequences are putatively functional, with no internal stop codons.

**Table 1:** γ-Gliadin sequences cloned from wheat and *Aegilops *species

Species	Accession	Genome formula	Sequenced colonies	Putatively functional (Genbank No.)	Pseudogene (Genbank No.)	Total
*T. aestivum*	Chinese Spring	AABBDD	41	25 (FJ006589–FJ006613)	4 (FJ006678–FJ006681)	29
SHW	SHW-L1	AABBDD	20	10 (FJ006614–FJ006623)	2 (FJ006682–FJ006683)	12
*T. dicoccoides*	2--1	AABB	18	8 (FJ006563–FJ006570)	1 (FJ006675)	9
*T. dicoccoides*	3--1	AABB	18	6 (FJ006571–FJ006576)	1 (FJ006676)	7
*T. turgidum*	AS2255	AABB	33	12 (FJ006577–FJ006588)	1 (FJ006677)	13
*aegilopoides*	PI428001	A^m^A^m^	18	9 (FJ006624–FJ006632)	0	9
*monococcum*	PI191096	A^m^A^m^	18	1 (FJ006633)	10 (FJ006652–FJ006661)	11
*T. urartu*	PI428222	A^u^A^u^	18	7 (FJ006634–FJ006640)	7 (FJ006662–FJ006668)	14
*Ae. speltoides*	PI449338	SS	15	11 (FJ006692–FJ006702)	0	11
*Ae. bicornis*	CIae 47	S^b^S^b^	15	10 (FJ006703–FJ006712)	2 (FJ006669–FJ006670)	12
*Ae. longissima*	PI 604104	S^l^S^l^	15	11 (FJ006641–FJ006651)	1 (FJ006671)	12
*Ae. searsii*	PI 599123	S^s^S^s^	15	8 (FJ006684–FJ006691)	2 (FJ006672–FJ006673)	10
*Ae. sharonesis*	CIae 32	S^sh^S^sh^	15	9 (FJ006713–FJ006721)	1 (FJ006674)	10
*Ae. tauschii*	AS60	DD	18	11 (FJ006552–FJ006562)	0	11

Total			277	138	32	170

*T. aestivum *originated from the hybridization between *T. turgidum *and *Ae. tauschii*, followed by genome duplication. *T. turgidum *evolved from *T. dicoccoides *that was formed in the intercrossing between *T. urartu *and *Ae. speltoides *(closely relates to the B genome). *T. monococcum *was one representative of the A genome as well. The other four species of *Sitopsis *were used as relatives to *Ae. speltoides*; SHW = synthetic hexaploid wheat; *aegilopoides *= *T. monococcum *ssp. *aegilopoides*; *monococcum = T. monococcum *ssp. *monococcum*.

### Sequence polymorphism

ORF lengths of these sequences range from 678 to 1089 bp. It is notable that FJ006717 amplified from *Ae. sharonesis *is the shortest γ-gliadin gene so far reported (678 bp), whose repetitive region has 46 amino acid residues. FJ006692 and FJ006695 isolated from *Ae. speltoides *are the longest (1089 bp), containing 179 amino acid in their repetitive regions.

ORF lengths of the sequences derived from *Ae. sharonesis *are the most variable (678–1020 bp), while the sequences from *T. monococcum *ssp.*aegilopoides *are the most conserved in length. Lengths of the repetitive region (domain II) of these sequences vary greatly as well (138–537 bp) (Table 2). The lengths of ORF and the repetitive region were analyzed for difference on average. It is shown that they have a similar changing trend. Considering the sequence alignment result, it could be concluded that the repetitive region is mainly responsible for the size heterogeneity of the γ-gliadins.

**Table 2:** Length polymorphisms of γ-gliadin sequences

Species	Range of ORF length	Average^1^	SD^1^	Range of Domain II length	Average^2^	SD^2^
*T. aestivum*	684–984 bp (300 bp)	857.8 bp	77.4654	183–447 bp (264 bp)	348.3 bp	72.5809
SHW	732–921 bp (189 bp)	859.6 bp	48.4814	198–408 bp (210 bp)	353.6 bp	52.8423
*T. dicoccoides*	729–1047 bp (318 bp)	967.6 bp	112.382	228–504 bp (276 bp)	436.1 bp	99.6129
*T. turgidum*	786–909 bp (123 bp)	858.0 bp	24.8478	288–408 bp (120 bp)	349.8 bp	25.5305
*aegilopoides*	858–864 bp (6 bp)	858.7 bp	2.0000	366–357 bp (9 bp)	358.0 bp	3.0000
*monococcum*	857–873 bp (16 bp)	859.6 bp	4.6103	356–375 bp (19 bp)	329.0 bp	5.4855
*T. urartu*	753–903 bp (150 bp)	846.9 bp	31.8889	252–360 bp (108 bp)	343.1 bp	27.9600
*Ae. speltoides*	909–1089 bp (180 bp)	941.7 bp	72.8136	408–537 bp (129 bp)	431.5 bp	52.1831
*Ae. bicornis*	908–999 bp (91 bp)	940.4 bp	39.3041	407–456 bp (49 bp)	417.9 bp	14.8352
*Ae. longissima*	690–957 bp (267 bp)	874.3 bp	77.9943	138–408 bp (270 bp)	343.8 bp	88.5100
*Ae. searsii*	720–926 bp (206 bp)	890.6 bp	60.3401	219–425 bp (206 bp)	386.3 bp	60.8515
*Ae. sharonesis*	678–1020 bp (342 bp)	857.4 bp	131.601	138–468 bp (330 bp)	315.2 bp	132.9000
*Ae. tauschii*	903–1047 bp (144 bp)	1015.1 bp	56.4490	363–504 bp (141 bp)	476.5 bp	49.0171

Total	678–1089 bp (411 bp)			138–537 bp (399 bp)		

ORF refers to the sequences from initial codon to mature stop codon; Average^1 ^= the average length of ORF; SD^1 ^= standard deviation of ORF length; Average^2 ^= the average length of Domain II; SD^2 ^= standard deviation of the length of Domain II; SHW = synthetic hexaploid wheat; *aegilopoides *= *T. monococcum *ssp. *aegilopoides*; *monococcum = T. monococcum *ssp. *monococcum*.

Table 3 shows the three estimates of genetic diversity. Haplotype diversity is very high, with a range from 0.909 ± 0.079 in *Ae. longissima *to 1.000 ± 0.030 in *T. turgidum*. Estimates of π vary from 0.01522 ± 0.00981 in *T. monococcum *ssp. *monococcum *to 0.08129 ± 0.00484 in *T. aestivum*. Estimates of θw range from 0.02584 ± 0.00336 in *T. monococcum *ssp. *aegilopoides *to 0.08053 ± 0.00584 in *T. dicoccoides*. θw and π show different trends in the rank of their values.

**Table 3:** Nucleotide diversity, neutrality test and minimum number of recombination events in wheat and *Aegilops *species

Species	No.	S	Mut	π ± SD	θ w ± SD	H	Hd ± SD	Tajima's D	Fu and Li's D	ZnS	Rm
*T. aestivum*	29	154	180	0.08129 ± 0.00484	0.07053 ± 0.00568***	25***	0.983 ± 0.017***	-0.05382	0.59109	0.1109***	19
SHW	12	130	139	0.06341 ± 0.01143	0.06321 ± 0.00554***	11***	0.985 ± 0.040***	-0.28955	0.4752	0.2358***	6
*T. dicoccoides*	16	190	212	0.06635 ± 0.01591	0.08053 ± 0.00584***	12***	0.917 ± 0.064***	-1.14004	-1.12294	0.2222***	14
*T. turgidum*	13	148	160	0.06497 ± 0.01063	0.06300 ± 0.00518***	13***	1.000 ± 0.030***	-0.21148	-0.0347	0.2543***	9
*aegilopoides*	9	59	60	0.01703 ± 0.00991	0.02584 ± 0.00336***	8***	0.972 ± 0.064***	-1.80203*	-2.05794*	0.7418***	0
*monococcum*	11	71	71	0.01522 ± 0.00981	0.02859 ± 0.00339***	10***	0.982 ± 0.046***	-2.22668***	-2.74095**	0.6944***	0
*T. urartu*	14	82	85	0.02016 ± 0.00659	0.03499 ± 0.00386***	13***	0.989 ± 0.031***	-1.97542*	-2.53161*	0.2756***	2
*Ae. speltoides*	11	116	117	0.03998 ± 0.01801	0.04371 ± 0.00406***	9***	0.945 ± 0.066***	-0.44816	1.13453	0.8323***	2
*Ae. bicornis*	12	141	146	0.06499 ± 0.00980	0.05318 ± 0.00448***	11***	0.985 ± 0.040***	0.84437	0.02362	0.4440***	1
*Ae. longissima*	12	99	107	0.06477 ± 0.00878	0.05331 ± 0.00536***	9***	0.909 ± 0.079***	0.57962	0.72021	0.2949***	4
*Ae. searsii*	10	116	121	0.04333 ± 0.01678	0.05719 ± 0.00531***	8***	0.933 ± 0.077***	-1.35918	-1.34402	0.4010***	2
*Ae. sharonesis*	10	105	119	0.04776 ± 0.01564	0.05836 ± 0.00570***	8***	0.933 ± 0.077***	-1.37962	-1.35868	0.3258***	5
*Ae. tauschii*	11	109	114	0.03109 ± 0.01466	0.04478 ± 0.00429***	10***	0.982 ± 0.046***	-1.61405	-1.83433	0.5936***	1

SHW = synthetic hexaploid wheat; *aegilopoides *= *T. monococcum *ssp. *aegilopoides*; *monococcum = T. monococcum *ssp.* monococcum*; No. = the number of sequences; S = segregating sites; Mut = total number of mutations; SD = standard deviation; H = haplotypes; Hd = haplotype diversity; *indicates P < 0.05; **indicates P < 0.01; *** indicates P < 0.001.

Table 3 also shows the result of neutrality test. Tajima's D is negative in most species except for *Ae. bicornis *and *Ae. longissima*. Three species/subspecies of diploid wheat show a significant negative Tajima's D due to an excess of variants, which indicates that they significantly departure from an equilibrium neutral model. Fu and Li' D is negative among eight species. The same three species show a significant excess of mutations.

### Linkage disequilibrium (LD) and recombination

LD between γ-gliadin sequences in different genomes could be estimated by ZnS, which has a range from 0 (equilibrium) to 1 (disequilibrium). The values of ZnS are in a wide range (Table 3). The highest is 0.7418 in *T. monococcum *ssp. *aegilopoides*, and the lowest is 0.1109 in *T. aestivum*. However, there is a high level of LD in γ-gliadin family. A measure of the minimum number of recombination events for these data yields a range of 0–19.

### Repetitive region

The repetitive domain is long (encompassing about 45% of total γ-gliadins) and consists of regular short repeats (see Additional file 1). Multiple sequence alignment indicates that the two end of this domain are conserved, and the changes mainly caused by SNP (single nucleotide polymorphism). The internal part is highly variable, mainly resulting from repeat insertions/deletions. These repeat domains have been evolving rapidly, and should not be used as a basis for determining relatedness [19]. To investigate whether the repetitive regions are related to the genomic origin of γ-gliadin genes, the nucleotide sequences of this domain were used to carry out the phylogenic analysis. The great majority of the sequences from diploid wheat and *Aegilops *species show a clustering according to their genomic origins (data not shown). Therefore, it would be much better if the repetitive domains were used in the relatedness analysis.

The phylogenic analysis facilitates us to array peptide repeat pattern in parallel. The reported typical unit of γ-gliadins is PFPQ_1–2_(PQQ)_1–2 _[19]. Our consensus repeat motif is similar to it, and modified by the substitution of several residues, i.e. **P**(Q/L/S/T/I/V/R/A)**F**(S/Y/V/Q/I/C/L)**P**(R/L/S/T/H/C/Y)**Q**_1–2_**(P**(S/L/T/A/F/H)**QQ)**_1–2_. The initial and final repeat motifs do not fit the consensus motif but are included since they appear to be related to the consensus [19]. The unit is repeated from 7 to 22 times and interspersed by additional residue(s).

### Relatedness of γ-gliadin genes

**Figure 2:**
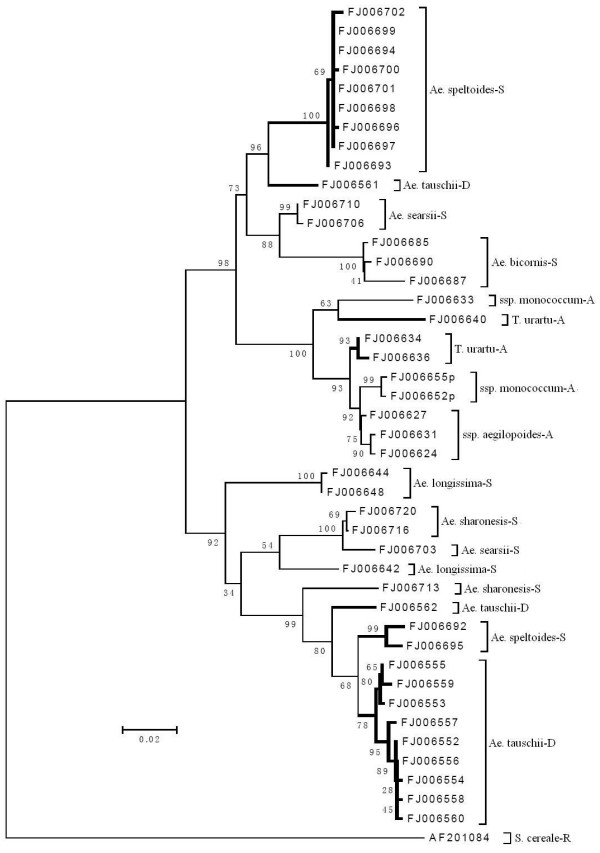
**Neighbour-joining tree of the γ-gliadin sequences from diploid wheat and *Aegilops *species**. Wider lines indicate the nodes of the three subtrees in Additional File 2; 'p' = pseudogene; AF20184 is the outgroup, which is a 75 K γ-secaline gene.

Firstly, the γ-gliadin genes cloned from *T. urartu*, *Ae. speltoides *and *Ae. tauschii *were used to carry out the phylogenic analysis (see Additional File 2). It contains three subtrees and shows a clear clustering of these γ-gliadin genes according to their genomic origins. Exceptions are some genes from *Ae. speltoides *and *Ae. tauschii*. Secondly, to gain a further insight into the relationship between γ-gliadin genes from different genomes, we included the γ-gliadin sequences from the two subspecies of *T. monococcum *and other four *Sitopsis *species i.e. *Ae. sharonesis*, *Ae. bicornis*, *Ae. longissima *and *Ae. searsii *(Table 1) into the phylogenic analysis. They cover all the diploid species that represent the ancestral genomes of *T. aestivum*. Inclusion of these sequences does not alter the fundamental structure of the phylogenic tree, and continues to strongly support the division of the three subtrees. For a clear arrangement, we have chosen all the γ-gliadin genes from *Ae. speltoides *and *Ae. tauschii*, and randomly selected three sequences from each of the other diploid species to create a phylogenic tree (Figure 2). The presence of strongly supported mixed subtrees indicates that there is no obvious discrimination between *Gli-D1 *and *Gli-S1 *loci, compared with *Gli-A1*. Furthermore, the sequences from the five *Sitopsis *species could not be clearly distinguished in the two subtrees.

To assess the phylogenic relationship among the γ-gliadin genes from tetraploid wheat, we aligned the sequences of diploid and tetraploid species, and constructed dendrograms. The γ-gliadin sequences that clustered together with the sequences from diploid wheat (96% bootstrap; cluster I) should be closely related to the A genome (see Additional File 3). The sequences that fall in cluster II are supposed to locate at the *Gli-B1 *locus of tetraploid wheat. It is interesting that the eleven γ-gliadin sequences of *T. dicoccoides *exactly clustered together with those of *Ae. tauschii *in a small cluster (98% bootstrap), which confirms the close relationship between the *Gli-S1 *(*Gli-B1*) and *Gli-D1 *loci. Using the characteristics of clusters described above, we could only identify the sequences relating to the A genome (cluster I; see Additional File 4) among those cloned from hexaploid wheat.

### Cysteine and Classification

**Figure 3:**
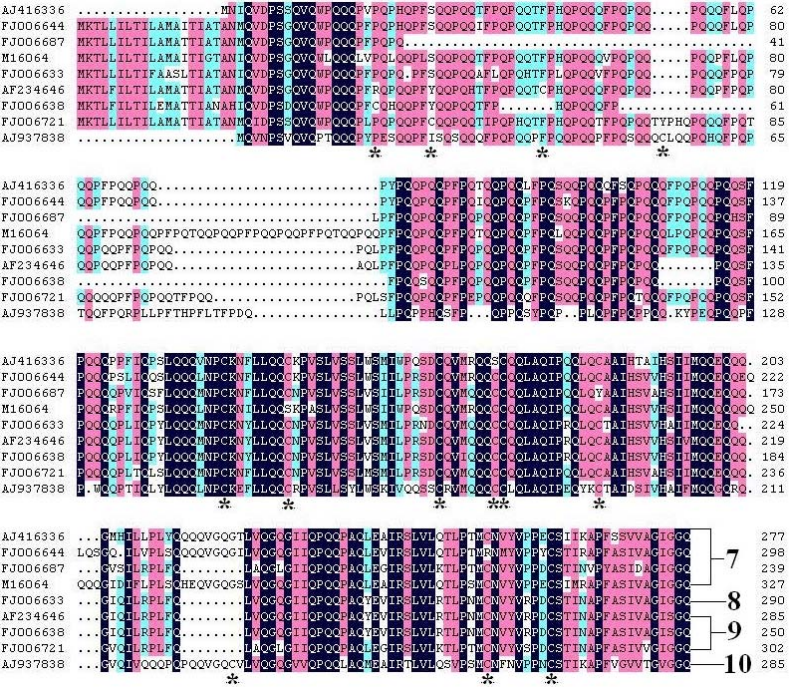
**Alignment of nine γ-gliadin polypeptide sequences, which are representatives of each cysteine distribution pattern**. The gaps in the internal parts of the repetitive domain are not definitely addressed. Asterisks at the bottom of alignment indicate the positions of cysteine. The numbers at the end indicate the number of cysteine residues in each sequence. AJ937838^a^, AF234646^a^, FJ006638^a^, FJ006721^a^, FJ006633^a^, AJ416336^a^, M16064^a^, FJ006687^a^ and FJ006644^a^ are representatives of the patterns P1 to P9, respectively (Table 5).

The deduced amino acid peptides of the 169 putatively functional γ-gliadin genes (Table 1 and Table 4) were analyzed for differences in the number and placement of cysteine residues [12,19-28]. Most of the peptides (116/169) contain eight cysteine residues that form four intramolecular disulphide bonds [29]. The eight cysteine residues are located following a conserved pattern (Figure 1) [19], where the fourth and fifth cysteines are consecutive in the polypeptide chain. Fifty-three γ-gliadins show modified patterns of cysteine distribution either by adding/deleting cysteine residues or changing their relative locations (Table 5; Figure 3), among which 1, 45 and 7 peptides contain 10, 9 and 7 cysteine residues, respectively. Differences in the number of cysteine among γ-gliadins are likely to result from point mutations, which involve CGC-TGC, TCC-TGC, TAC-TGC, TTC-TGC, GGT-TGT and TCG-TGC. Besides the number of cysteine, the main difference among the nine patterns resides in the presence of the first cysteine residue either in the non-repetitive region or nearer along in the repetitive domain. It is notable that AJ937838 has an additional cysteine in the glutamine-rich region.

**Table 4:** Putatively functional γ-gliadin genes registered in public database

Accession	Species	Cultivar	Molecular Type	Reference
AF120267	T. aestivum ssp. spelta	Oberkulmer	Genomic DNA	[20]
AF144104	*T. aestivum*	Forno	Genomic DNA	[20]
AF234643	*T. aestivum*	Cheyenne	mRNA	[19]
AF234644	*T. aestivum*	Cheyenne	mRNA	[19]
AF234646	*T. aestivum*	Cheyenne	[15]	[19]
AF234647	*T. aestivum*	Cheyenne	Genomic DNA	[19]
AF234649	*T. aestivum*	Cheyenne	Genomic DNA	[19]
AF234650	*T. aestivum*	Cheyenne	mRNA	[19]
AJ133613	*T. aestivum*	Yamhill	Genomic DNA	[21]
AF175312	*T. aestivum*	Yamhill	Genomic DNA	[21]
AJ416336	*T. aestivum*	Mjoelner	Genomic DNA	[22]
AJ416337	*T. aestivum*	Mjoelner	Genomic DNA	[22]
AJ416338	*T. aestivum*	Mjoelner	Genomic DNA	[22]
AJ416339	*T. aestivum*	Mjoelner	Genomic DNA	[22]
AJ937838	*T. aestivum*	Neepawa	mRNA	
AY338386	*T. aestivum*	Anza	mRNA	[12]
AY338387	*T. aestivum*	Anza	mRNA	[12]
AY338388	*T. aestivum*	Bobwhite	mRNA	[12]
AY338389	*T. aestivum*	TB175	mRNA	[12]
AY338390	*T. aestivum*	TB236	mRNA	[12]
AY338391	*T. turgidum *ssp.*durum*	TD92	mRNA	[12]
AY338392	*T. turgidum *ssp. *durum*	TD92	mRNA	[12]
AY338393	T. turgidum ssp. durum	TD1A23	mRNA	[12]
AY338394	*T. turgidum *ssp.*durum*	TD1A23	mRNA	[12]
AY338395	*T. turgidum *ssp.*durum *× *Hordeum Chilense*		mRNA	
D78183	T. aestivum	Hard red spring 1CW	Genomic DNA	[23]
EF151018	*T. aestivum*	Chinese Spring	mRNA	[24]
M13713	*T. aestivum*	Yamhill	Genomic DNA	[25]
M16064	*T. aestivum*	Yamhill	Genomic DNA	[26]
M36999	*T. aestivum*	Yamhill	Genomic DNA	[27]
X77963	*T. turgidum *ssp. *durum*	Lira	Genomic DNA	[28]

**Table 5:** Classification of γ-gliadins

Group	Pattern	Subgroup	GenBank No.					
C10	C10-P1	SG-1	AJ937838^a,T, L^					
C9	C9-P2	SG-2	AF234646					
	C9-P3	SG-3	FJ006638					
	C9-P4	SG-4	FJ006561	FJ006573^**T**^	FJ006605	FJ006622	FJ006693	FJ006694
			FJ006696	FJ006697	FJ006698	FJ006699	FJ006700	FJ006701
			FJ006702					
		SG-5	FJ006641	FJ006643	FJ006650	FJ006704	FJ006706	FJ006710
			FJ006711	FJ006712	FJ006721			
		SG-6	AF234647	FJ006576^**T**^	FJ006577	FJ006590	FJ006600	FJ006612^**T**^
			FJ006685	FJ006686	FJ006688	FJ006689	FJ006690	FJ006691
			FJ006592	M36999	X77963			
		SG-7	FJ006596	FJ006602	FJ006604^**T, L**^	FJ006617^**L**^	FJ006618	FJ006621
C8	C8-P5	SG-8	AF175312	AF234650	AY338388	FJ006552	FJ006553	FJ006554
			FJ006555	FJ006556	FJ006557	FJ006558	FJ006559	FJ006560
			FJ006562	FJ006563	FJ006564^**T**^	FJ006565	FJ006566	FJ006568
			FJ006569	FJ006570	FJ006571	FJ006572	FJ006574	FJ006575
			FJ006595	FJ006603	FJ006692	FJ006695	FJ006707	FJ006713
		SG-9	AF120267^**L**^	AF144104^**L**^	AY338389^**T, L**^	EF151018^**L**^	FJ006591^**T, L**^	
		SG-10	FJ006649^**T, L**^	FJ006703	FJ006705	FJ006708	FJ006709	FJ006714^**T, L**^
			FJ006715^**L**^	FJ006716^**T, L**^	FJ006717^**T**^	FJ006718^**L**^	FJ006719^**T, L**^	FJ006720^**L**^
		SG-11	FJ006642^**T, L**^					
		SG-12	AF234649	AJ133613	AY338386^**T, L**^	AY338387^**T, L**^	AY338390	AY338391^**T, L**^
			AY338393^**L**^	AY338394^**T, L**^	AY338395^**T**^	D78183	FJ006567^**T, L**^	FJ006578^**T, L**^
			FJ006585^**T, L**^	FJ006588^**T, L**^	FJ006599	FJ006607^**T, L**^	FJ006619^**T, L**^	FJ006645^**L**^
			FJ006646^**L**^	FJ006647^**L**^	FJ006648^**L**^	FJ006651	FJ006684^**T, L**^	M13713^**L**^
		SG-13	AF234643^**T, L**^	AF234644	AY338392^**T**^	FJ006579	FJ006580	FJ006581
			FJ006582	FJ006583	FJ006584	FJ006586	FJ006587	FJ006589
			FJ006593	FJ006594	FJ006597	FJ006598^**T**^	FJ006601	FJ006606
			FJ006608^**T**^	FJ006609	FJ006610	FJ006611	FJ006613	FJ006614
			FJ006615	FJ006616	FJ006620	FJ006623	FJ006624	FJ006625
			FJ006626	FJ006627	FJ006628	FJ006629	FJ006630	FJ006631
			FJ006632	FJ006633	FJ006634	FJ006635^**L**^	FJ006636	FJ006637
			FJ006639	FJ006640				
C7	C7-P6	SG-14	AJ416336^**L**^	AJ416337^**T, L**^	AJ416338^**L**^	AJ416339^**L**^		
	C7-P7	SG-15	M16064					
	C7-P8	SG-16	FJ006687^**T**^					
	C7-P9	SG-17	FJ006644^**L**^					

The ^a^ indicate the representatives of the nine patterns used in Figure 3; '^**T**^' and '^**L**^' indicate the sequences that contain a higher proportion of total essential amino acids and Lys, respectively. Some sequences are marked more than once. For example, AJ937838^**T, L **^means AJ937838 has been used in Figure 3, and the proportions of its total essential amino acids and Lys are higher.

Using the number of cysteine residues as a discrimination factor, we classified the 169 putatively functional γ-gliadin genes into four groups, i.e. C7, C8, C9 and C10 (Table 5). Forty-five sequences of C9 share a high identity (88.93%). A comparison of the relative position of cysteine residues within each group allows to define respectively four, one, three and one cysteine distribution patterns within C7, C8, C9 and C10 (Figure 3). Those patterns with fewer than five members are directly recognized as subgroups (Table 5). Phylogenic analyses of the deduced mature proteins of the members of pattern C9-P4 and C8-P5 indicate that C9-P4 and C8-P5 should be grouped into four and six subgroups, respectively (see Additional Files 5 and 6). Accordingly, we have totally defined 17 γ-gliadin subgroups. The frequencies of the 17 subgroups among the wheat and *Aegilops *accessions (Table 1) are shown in Additional file 7. The γ-gliadins of each accession prefer to fall into one or two subgroups.

### Amino acid composition

The proportion of each amino acid differs significantly in γ-gliadins. γ-Gliadins are rich in Gln (33.40%) and Pro (16.64%), followed by Leu (6.59%), and show a 20:10:3 ratio of Q:P:F for total amino acids. Essential amino acids are indispensable for good health, but cannot be synthesized in the body and must be supplied in the diet. There are eight (Trp, Lys, Met, Thr, Phe, Val, Ile and Leu) out of the 20 naturally occurring amino acids considered essential for humans. One of the problems with wheat flour is that they do not provide enough essential amino acids, which is mainly caused by the lack of essential amino acids in seed storage proteins. We analyzed the proportions of the eight essential amino acids in each of the deduced mature γ-gliadin peptides. A wide range of the proportion of each essential amino acid has been observed. The limiting amino acid (the essential amino acid found in the smallest quantity in the foodstuff) in wheat is Lys. Its average proportion is 0.69%, with a range of 0.29–1.90%. The values are a little higher than those of Trp (average 0.65%; range 0.00–1.75%). The average proportions of Met, Thr, Phe, Val, Ile and Leu are 1.93%, 2.38%, 4.95%, 5.04%, 5.39% and 6.59%, respectively, which range from 1.10–2.88%, 0.83–4.24%, 2.38–6.01%, 3.89–7.37%, 3.51–7.56% and 5.30–9.13%, respectively.

The repetitive domain is long and variable, consisting mostly of Gln (48.10%), Pro (27.53%) and Phe (9.36%). This domain has a different Q:P:F ratio (5:3:1). To provide more information for wheat breeding, we analyzed the essential amino acid proportions of mature γ-gliadin peptides while ignoring the repetitive domain (The nucleotide sequences left are about 530 bp). The average proportions of six out of the eight essential amino acids significantly increase up (see Additional file 8; t test, P < 0.05). Exceptions are Thr and Phe. Eighteen γ-gliadin sequences (belonging to nine subgroups) whose repetitive domains contain fewer than 85 amino acid residues (Table 6) were selected for additional comparison against the whole group. Compared with the other two sets of values (see Additional file 8), theirs are intermediate.

**Table 6:** γ-gliadin sequences whose repetitive domains contain fewer than 85 amino acid residues

Sequence	Number	ORF Length	Origin	Subgroup
AF234643	80	771 bp	*T. aestivum*	SG-13
AY338392	78	768 bp	*T. turgidum *ssp.*durum*	SG-13
FJ006564	80	783 bp	*T. dicoccoides*	SG-8
FJ006576	76	729 bp	*T. dicoccoides*	SG-6
FJ006591	73	744 bp	*T. aestivum*	SG-6
FJ006598	71	714 bp	*T. aestivum*	SG-13
FJ006607	83	783 bp	*T. aestivum*	SG-12
FJ006608	61	684 bp	*T. aestivum*	SG-13
FJ006612	76	729 bp	*T. aestivum*	SG-6
FJ006619	66	732 bp	SHW	SG-12
FJ006638	84	753 bp	*T. urartu*	SG-3
FJ006642	61	735 bp	*Ae. longissima*	SG-11
FJ006649	46	690 bp	*Ae. longissima*	SG-10
FJ006687	73	720 bp	*Ae. searsii*	SG-16
FJ006714	46	681 bp	*Ae. sharonesis*	SG-10
FJ006716	73	762 bp	*Ae. sharonesis*	SG-10
FJ006717	46	678 bp	*Ae. sharonesis*	SG-10
FJ006719	56	711 bp	*Ae. sharonesis*	SG-10
Average	68.3	731.5 bp		

Number = the number of amino acid residues in the repetitive domains.

Compared to the essential amino acid proportions of the sequences with a long repetitive domain, the most γ-gliadins with fewer than 85 residues in the repetitive domain contain a higher proportion of total essential amino acids and Lys (see Additional file 9). However, it is noticeable that the corresponding proportions of some γ-gliadins with a long repetitive domain are high as well. We determined the corresponding subgroups of each γ-gliadin (either total proportion of essential amino acid ≥ 29% or the amount of Lys ≥ 1%). Analysis indicated that these γ-gliadins tend to be the members of certain groups, such as SG-10 and SG-12 (Table 5).

### Analysis of CD toxic epitopes in γ-gliadins

The perfect matches in the 169 putatively functional genes to the three CD-toxic epitopes of γ-gliadins [17] are shown in Table 7. Each epitope appears at most once in every sequence, and all of them located at the repetitive domain. The result indicates that those subgroups or species contribute differently to the epitope content. Firstly, more γ-gliadins from *T. aestivum*, *Ae. longissima *and *Ae. bicornis *contain CD toxic peptides. Secondly, 23 sequences altogether contain these epitopes. The epitopes are present in seven subgroups (SG-7 (ratio = 2/6); SG-9 (5/5); SG-10 (4/12); SG-12 (5/34); SG-13 (2/44); SG-14 (4/4) SG-17 (1/1)). However, a majority of them (16/23) are members of C8. Five out of seven members of C7 contain the toxic peptides. Only two out of the 45 sequences of C9 contain the toxic epitopes. One (FJ006591) of the 18 γ-gliadins with fewer than 85 residues in the repetitive domain contain a toxic epitope. Finally, occurrences of the three epitopes are at the frequencies of 18/169, 19/169 and 11/169, respectively.

**Table 7:** The distribution of three T cell stimulatory epitopes in γ-gliadins

FLQPQQPFPQQPQQPYPQQPQQPFPQ			LQPQQPFPQQPQQPYPQQPQ			FSQPQQQFPQPQ		
Gene	Subgroup	Species	Gene	Subgroup	Species	Gene	Subgroup	Species
AF120267	SG-9	*T. spelta*	AF120267	SG-9	*T. spelta*	AF120267	SG-9	*T. spelta*
AF144104	SG-9	*T. asetivum*	AF144104	SG-9	*T. asetivum*	AF144104	SG-9	*T. asetivum*
AJ416336	SG-14	*T. asetivum*	AJ416336	SG-14	*T. asetivum*	AJ416336	SG-14	*T. asetivum*
AJ416339	SG-14	*T. asetivum*	AJ416337	SG-14	*T. asetivum*	AJ416337	SG-14	*T. asetivum*
AY338389	SG-9	*T. asetivum*	AJ416339	SG-14	*T. asetivum*	AJ416338	SG-14	*T. asetivum*
EF151018	SG-9	*T. asetivum*	AY338389	SG-9	*T. asetivum*	AJ416339	SG-14	*T. asetivum*
FJ006586	SG-13	*T. turgidum*	EF151018	SG-9	*T. asetivum*	AY338389	SG-9	*T. asetivum*
FJ006593	SG-13	*T. asetivum*	FJ006586	SG-13	*T. turgidum*	EF151018	SG-9	*T. asetivum*
FJ006644	SG-17	*Ae. Longissima*	FJ006593	SG-13	*T. asetivum*	FJ006591	SG-9	*T. asetivum*
FJ006645	SG-12	*Ae. Longissima*	FJ006644	SG-17	*Ae. longissima*	FJ006596	SG-7	*T. asetivum*
FJ006646	SG-12	*Ae. Longissima*	FJ006645	SG-12	*Ae. longissima*	FJ006604	SG-7	*T. asetivum*
FJ006647	SG-12	*Ae. Longissima*	FJ006646	SG-12	*Ae. longissima*			
FJ006648	SG-12	*Ae. Longissima*	FJ006647	SG-12	*Ae. longissima*			
FJ006684	SG-12	*Ae. Searsii*	FJ006648	SG-12	*Ae. longissima*			
FJ006703	SG-10	*Ae. Bicornis*	FJ006684	SG-12	*Ae. searsii*			
FJ006705	SG-10	*Ae. Bicornis*	FJ006703	SG-10	*Ae. bicornis*			
FJ006708	SG-10	*Ae. Bicornis*	FJ006705	SG-10	*Ae. bicornis*			
FJ006709	SG-10	*Ae. Bicornis*	FJ006708	SG-10	*Ae. bicornis*			
			FJ006709	SG-10	*Ae. bicornis*			

*T. spelta *= *T. aestivum *ssp. *spelta*; *T. turgidum*= *T. turgidum *ssp. *turgidum*

A further look at the sequences reveals that amino acid change(s) in particular epitopes caused by SNP disrupt the continuous peptides. Forty-one analogues of epitopes are shown in Additional file 10. Substitutions of amino acid often concern glutamine and proline residue. Amino acid insertions and deletions also occur frequently, which destroy the epitopes (data not shown).

## Discussion

### Diversity of γ-gliadin sequences

Sequence diversity between the γ-gliadin genes is due to SNPs and variations in the repetitive region, and the latter is mainly responsible for the size heterogeneity of the γ-gliadins [11,19]. It is comparatively confirmed by our result. Nucleotide diversity (θw and π) was estimated to be lower in self-pollinated diploid wheat than open-pollinated *Aegilops *species (Table 3). Besides the large number of segregating sites and mutations, haplotype diversity of γ-gliadin genes is high in every species investigated, which indicates a high nucleotide diversity of γ-gliadin family. Extensive linkage disequilibrium found with different species indicates similar ancestries between freely recombining portions of *Gli-1 *loci. Negative values of the neutrality test statistics (Tajima's D, Fu and Li's D) in most species suggest that they are mainly under negative selection. Overall, *Gli-1 *loci in different species are diverse, although γ-gliadin is supposed to be the most ancient family among prolamins [18].

### Evolution of γ-gliadin multigene family

To avoid PCR bias, two forward and two reverse primers are used to amplify the full ORF of γ-gliadin genes. As a result, we isolated 29 unique γ-gliadin genes from *T. aestivum *cv 'Chinese Spring'. Meanwhile, nine to 14 unique genes were cloned from the nine diploid wheat and *Aegilops *species, respectively, with an average of 11 (Table 1). There are 15 to 40 copies of γ-gliadin genes in Chinese Spring [10]. Considering the copy number, it can be concluded that the γ-gliadin sequences we cloned could represent the whole γ-gliadin family. Multiplication of γ-gliadin genes should have occurred in the diploid level, since a large number of γ-gliadin genes have been isolated from a few accessions, which is similar to the result on α-gliadin genes [30].

The number of γ-gliadin sequences cloned from two accessions of *T. dicoccoides *and AS2255 of *T. turgidum *are 9, 7 and 13, respectively. It seems likely that tetraploid wheat went through a bottleneck in *Gli-1 *loci, which is supported by the small copy numbers compared with those of common wheat and diploid species. We could conclude that great changes happened to the *Gli-1 *regions in the formation of tetraploid wheat. It is possible that some γ-gliadin sequences disappeared from the genome. It is interesting to note that similar event seems not occur during the formation of *T. aestivum*. Experimental data, based on the simulation of the evolutionary step by synthetic hexaploid wheat (SHW-L1) and its parental lines (*T. turgidum *accession AS2255 and *Ae. tauschii *accession AS60), supports the assumption of γ-gliadin sequences disappearance. Therefore, duplication and subsequent divergence might be important as well at the polyploid level in contrast to the diploid strains.

Pseudogenes are involved in phylogenic analysis, and they fall into correct clusters, which indicate that the occurrence of pseudogenes should have take place after the divergence of diploid species.

### Classification

It is the primary structure of peptides that finally determines very specific properties of the ending biomaterials [31]. The structures are important to dough rheology and other aspects of food technology [32]. According to the characteristics of primary structure, i.e. number and placement of cysteine residues and the phylogenic result, we divided γ-gliadins into 17 subgroups based on the mature peptides (without signal peptide) (Table 5). The different subfamilies are very distinct from each other. The classification of γ-gliadins has essential importance with regard to dough quality, since cysteine residues play a critical role in unique properties of wheat flour. Typical γ-gliadins contain eight cysteine residues. We have also found γ-gliadins containing seven, nine and even ten cysteine residues. Furthermore, we identified nine cysteine distribution patterns. Changes in position and number of cysteine residues might affect the pattern of disulphide bond formation, resulting in failure of forming some intramolecular disulphide bond(s). These cysteine residues would then be available for intermolecular disulphide bond formation and polymer-building [33]. However, we have not known whether these gliadins are chain terminator (only one cysteine residue available for intermolecular disulphide) or chain extenders (subunits with more than one cysteine residues that form inter-molecular disulphide bonds), which would presumably have a negative effect on flour quality or allow the formation of stronger dough, respectively [34,35].

Alternatively, we classified γ-gliadins into two types: i.e. repetitive domain<85 amino acids and repetitive domain ≥ 85 amino acids, which are named as sequences with a short repetitive domain (18 sequences) and sequences with a long repetitive domain (151 sequences) respectively. The repetitive domain is rich in glutamine and proline, which is the major sequence variation that discriminates the different γ-gliadins.

### Nutritional quality

It is well known that nutritional quality of food that lack essential amino acids is low, as the body tends to convert the amino acids obtained into fats and carbohydrates. Therefore, a balance of essential amino acids is necessary for a high degree of net protein utilization (the mass ratio of amino acids converted to proteins: amino acids supplied). The net protein utilization is profoundly affected by the limiting amino acid proportion (the essential amino acid found in the smallest quantity in the foodstuff). The limiting amino acid of wheat is lysine, which mainly caused by a low level of lysine in gliadin [36], since gliadins account for about half of the total storage proteins [3]. We systematically analyzed the proportions of eight essential amino acids of γ-gliadins, which indicates that subgroup SG-10 and SG-12 and the γ-gliadins with a short repetitive domain contain higher proportions of lysine and total essential amino acids. A wide range of the proportion of each essential amino acid could be seen, which provides the possibility of breeding more nutritional wheat varieties.

### Perspective for wheat breeding programs

The only efficient therapy for CD is a life-long gluten-free diet. Conceivably, a diet based on gluten from a wheat species that expresses no or few T-cell stimulatory gluten peptides should be equally well tolerated by the celiac patients and, importantly, also be beneficial for disease prevention [37,38]. It is also indicated that the genetic differences in gliadins might allow designing strategies for selection and breeding of less toxic wheat varieties [[30,37] and [38]]. Our results indicate that 23 out of the 169 putatively functional sequences contain γ-gliadin epitopes, and that γ-gliadins with a short repetitive domain almost contain no toxic epitopes, with the exception of FJ006591. Meanwhile, 22 sequences out of those with a long repetitive domain contain γ-gliadin epitopes. Obviously, the classification depending on the length of repetitive domain is reflected in the occurrence of toxic epitopes. CD-toxic peptides of γ-gliadins are only found in the repetitive domain, especially the internal part, which is highly variable in length. Those γ-gliadins with a short repetitive domain contain a brief internal part, which means that they are not/nearly not toxic to the population with celiac disease. The two subgroups SG-10 and SG-12, which show a relatively good nutritional quality, present four (4/12) and five (5/34) members containing epitopes. Therefore, it is suggested that the genes with a short repetitive domain are more nutritional and valuable. It is reported that stimulatory epitopes in α-gliadins from the D genome is the highest, compared to those from the A and B genome [30,38]. However, we have not found any epitope in the γ-gliadins from *Ae. tauschii *(Table 7).

## Conclusion

We systematically characterized the γ-gliadin multigene family in common wheat and its closely related tetraploid and diploid species. It is shown that γ-gliadin family is highly diverse. Phylogenic analyses indicate a more close relationship between the *Gli-1 *loci of the B(S) and D genomes. According to the differences in primary structure, we have classified γ-gliadins into 17 subgroups, which might reflect their differences in the contributions to the processing qualities of wheat flour. The γ-gliadins with a short repetitive domain are relatively more nutritional, since they contain a higher proportion of essential amino acids. Moreover, these short γ-gliadins almost contain no toxic epitopes. Therefore, it is possible to breed wheat varieties, the γ-gliadins of which are less, even non-toxic and more nutritional.

## Methods

### Plant materials and DNA extraction

*T. aestivum *cv. 'Chinese Spring' (CS) and its closely related wheat and *Aegilops *species (Table 1), one synthetic hexaploid wheat accession (SHW-L1; in the 3^rd^–4^th ^generations) and its parental *Ae. tauschii *(AS60) and *T. turgidum *ssp.*turgidum *(AS2255) lines were used.

Genomic DNA was extracted from leaves of single adult plants with a CTAB (Cetyltrimethylammonium bromide) protocol [39].

### Primer design

The PCR primers to isolate γ-gliadin genes from genomic DNA were designed on the conserved parts of the 5' and 3' flanking sequences of γ-gliadin genes retrieved from Genbank , which are listed as follows:

Forward1: 5'-TATTAGTTAACGCAAATCCACC/TATG-3'

Forward2: 5'-CTTCACACAACTAGAGCACAAG-3'

Reverse1: 5'-GATGAATCAGCTAAGCAACGATG-3'

Reverse2: 5'-TCGTTACATCTATTGGTGCATCAG)'-3'

### PCR based gene cloning

PCR amplification was conducted in a 25 μl volume, consisting of 100 ng genomic DNA, 100 μM of each dNTPs, 1.5 mM of Mg^2+^, 2 pmol of each of the four primers, 0.75 U *Taq *polymerase with high fidelity (*TianGen*; P.R. China) and 2.5 μl 10×buffer (supplied with the *Taq *polymerase). The reactions were run in a PTC-240 (MJ Research, USA) thermal cycler with following program: an initial step of 94°C for 4 min; 35 cycles of 94°C for 45 sec, 57°C for 1 min and 72°C for 80 sec; then a final step of 8 min at 72°C.

The amplified products were separated in 1% agarose gel. The desired fragments were recovered and cloned into pMD-18T vector (*Takara*), then transformed into competent *E. coli *(JM109) cells. Positive colonies were screened out and sequenced by commercial company (*Invitrogen*).

### DNA sequence analysis

Sequenced clones were confirmed by Blast analysis , and aligned using DNAman (version5.2.2; Lynnon Biosoft), Clustal X (version 1.81) [40] and MEGA (version 3.1) [41]. Further modifications to the alignment were done manually. Bootstrap test of phylogenies (1000 replicates; neighbor-joining method) were carried out using MEGA for the nucleotide sequences from initial codon (ATG) to mature stop codon (TGA), on the basis of Kimura 2-parameter distances, complete deletion of gaps. Neighbor-joining trees (1000 replicates) were also constructed for classification of mature proteins on the basis of poisson correction, complete deletion of gaps.

Nucleotide diversity was estimated with three approaches. Indels were excluded from the estimates. The first method used the number of haplotypes to estimate heterozygosity [42]. The second approach used the average number of nucleotide differences per site between two sequences (π) [42]. The last method used the number of segregating sites to estimate nucleotide diversity per site (θw) [42]. The strength of linkage disequilibrium (LD) was estimated using the *Z*nS statistic [43], which is the average of r^2 ^(squared correlation coefficient) [44] over all pairwise comparisons. Furthermore, Sequence data analysis (minimum number of recombination events (Rm) [45] and a statistical test of neutrality (Tajima's D and Fu and Li's D) [46,47]) were performed as well. Coalescent simulations were used to test for significant differences [48,49]. DnaSP version 4.50.3 [50] software package was used to complete these analyses.

### Amino acid composition analysis

Amino acid composition data of mature γ-gliadin peptides were determined by MEGA. Statistical analyses were carried out by Statistica version 6.0 .

### Epitope screening

The program MEGA was used for matching the γ-gliadin epitopes. Only perfect matches were considered.

## Authors' contributions

PFQ, YMW and YLZ designed the experiments. PFQ selected the plant materials and designed the primers. PFQ, QC and XT cloned the γ-gliadin sequences. PFQ, YMW, TO and YLZ analyzed the sequences. PFQ, YMW and TO drafted the manuscript. All authors have read and approved the final manuscript.

## Supplementary Material

Additional file 1 **Alignment of the nucleotide sequences of the 170 γ-gliadin genes shown in Table **1. Click here for file

Additional File 2 **Phylogenic tree of the γ-gliadin gene sequences isolated from *T. urartu*, *Ae. speltoides *and *Ae. tauschii***. 'p' = pseudogene; T. urartu-A, Ae. speltoides-S and Ae. tauschii-D indicate species-genome; AF20184 is a 75 K γ-secaline gene, which is used as the outgroup. Click here for file

Additional File 3 **Phylogenic tree obtained from alignment of the γ-gliadin genes from tetraploid wheat.** Click here for file

Additional File 4 **Phylogenic tree obtained from alignment of the γ-gliadin genes from hexaploid wheat**. Cluster II contained the γ-gliadin genes from both *Gli-B1 *and *Gli-D1 *loci. Click here for file

Additional File 5 **Phylogenic analysis of the deduced mature proteins of the γ-gliadins in pattern C9-P4**. SG-4 to SG-7 = subgroups described in Table 5. Click here for file

Additional File 6 **Neighbour-joining tree of the γ-gliadins in pattern C8-P5, on the basis of alignment of the deduced mature protein sequences**. SG-8 to SG-13 = subgroups described in Table 5. Click here for file

Additional file 7 **The frequencies of the 17 subgroups among the wheat and Aegilops accessions used in Table**1. Click here for file

Additional file 8 **The average proportions of eight essential amino acids**. Avg-1, Avg-2 and Avg-3 indicate the average proportions of the eight essential amino acids in the 169 mature γ-gliadins (putatively functional), in the 18 γ-gliadins whose repetitive domains contain fewer than 85 amino acid residues (Their repetitive domains are included for data analyses; Table 6) and in the 169 mature γ-gliadins ignoring the repetitive domains, respectively. Data are mean ± SD (standard deviation). Click here for file

Additional file 9 **The γ-gliadins with higher proportions of Lys or total essential amino acids**. In bold are the γ-gliadins with fewer than 85 residues in the repetitive domain. Click here for file

Additional file 10 **Analogues of CD-toxic epitopes in γ-gliadins**. The bold and italic letters indicate amino acid substitutions. Click here for file
