# Supplementary figures and images for: The γ-gliadin multigene family in common wheat (Triticum aestivum) and its closely related species
Source: BMC Genomics. 2009 Apr 21;10:168. doi: 10.1186/1471-2164-10-168 (PMC2685405; doi:10.1186/1471-2164-10-168)

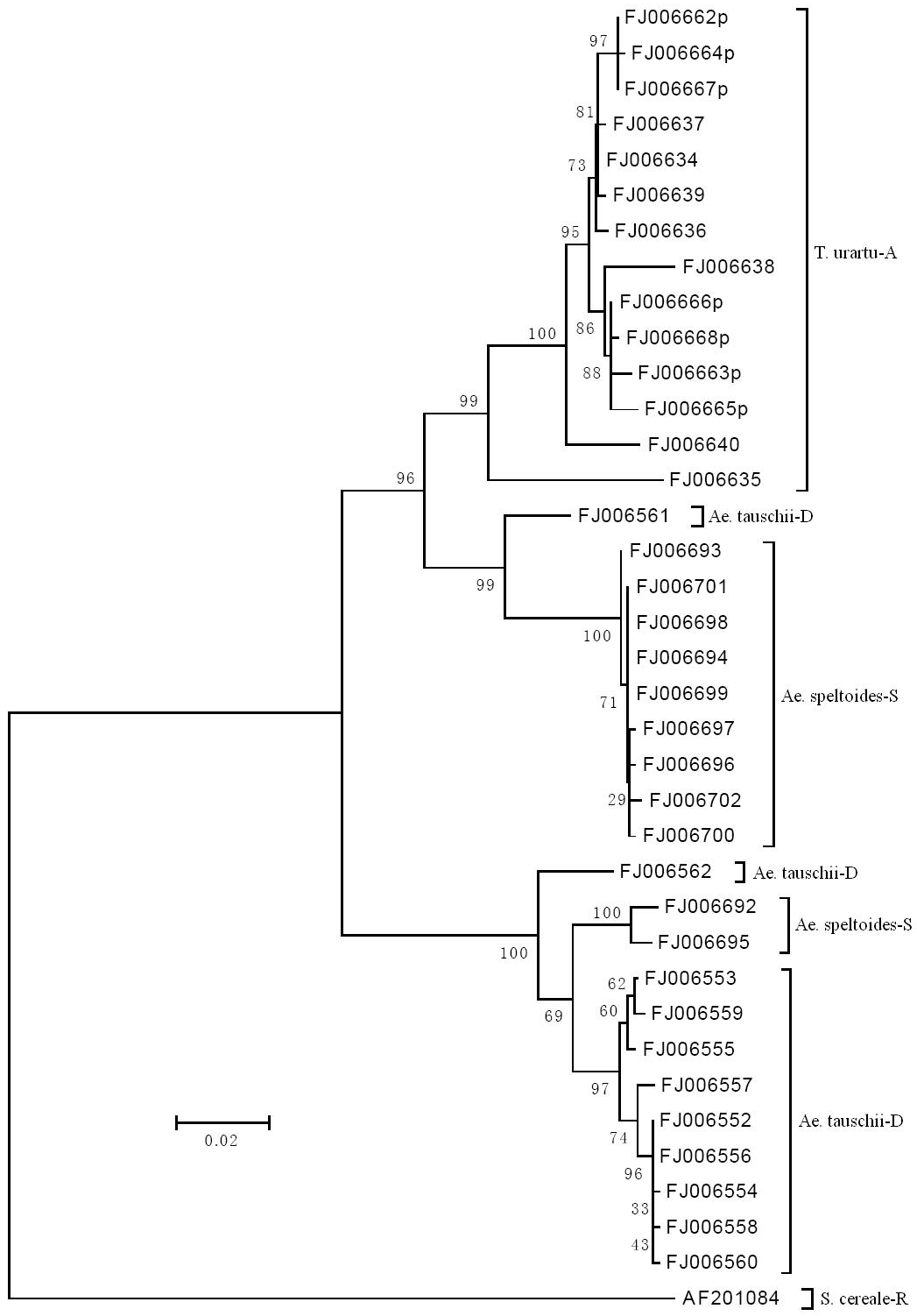

Supplement: Additional File 2 — Phylogenic tree of the γ-gliadin gene sequences isolated from T. urartu, Ae. speltoides and Ae. tauschii. 'p' = pseudogene; T. urartu-A, Ae. speltoides-S and Ae. tauschii-D indicate species-genome; AF20184 is a 75 K γ-secaline gene, which is used as the outgroup. [file 1471-2164-10-168-S2.png]

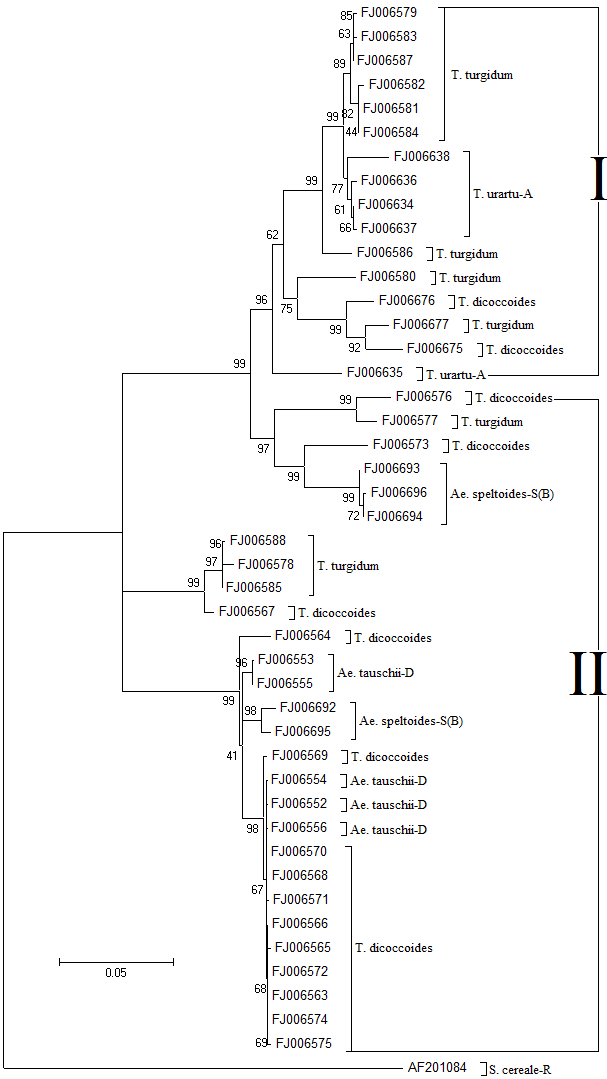

Supplement: Additional File 3 — Phylogenic tree obtained from alignment of the γ-gliadin genes from tetraploid wheat. [file 1471-2164-10-168-S3.png]

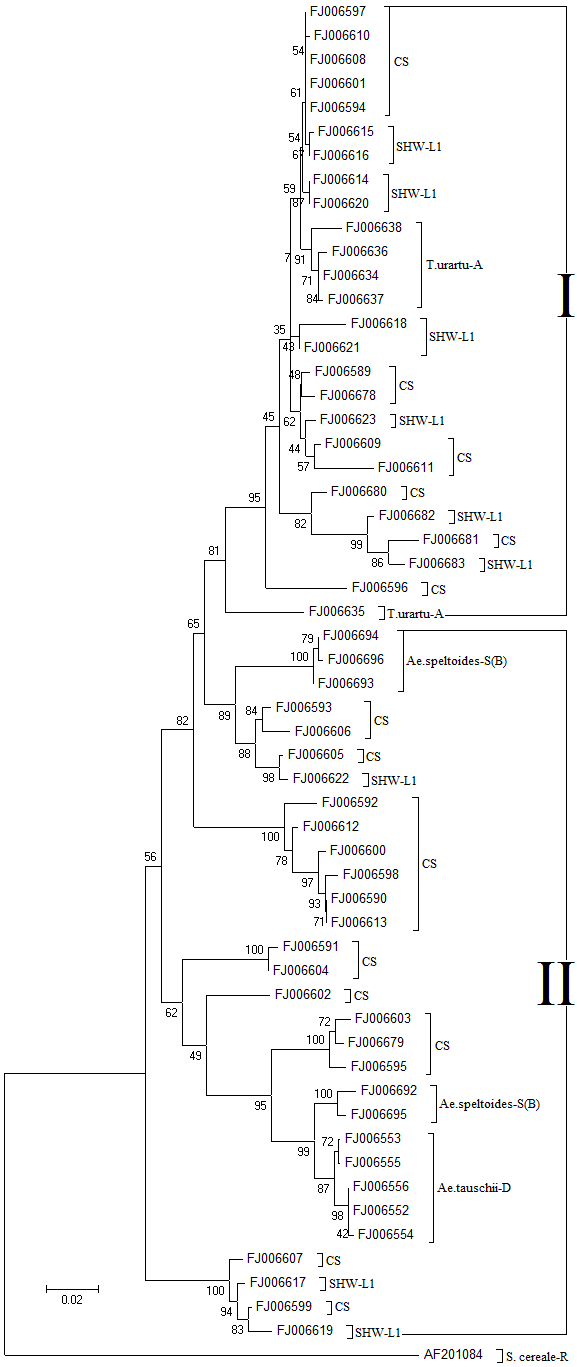

Supplement: Additional File 4 — Phylogenic tree obtained from alignment of the γ-gliadin genes from hexaploid wheat. Cluster II contained the γ-gliadin genes from both Gli-B1 and Gli-D1 loci. [file 1471-2164-10-168-S4.png]

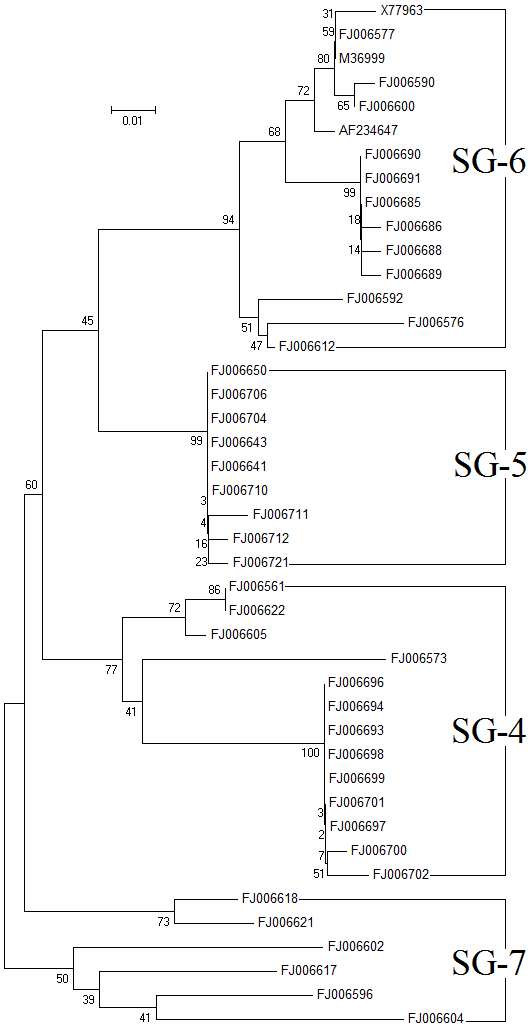

Supplement: Additional File 5 — Phylogenic analysis of the deduced mature proteins of the γ-gliadins in pattern C9-P4. SG-4 to SG-7 = subgroups described in Table 5. [file 1471-2164-10-168-S5.png]

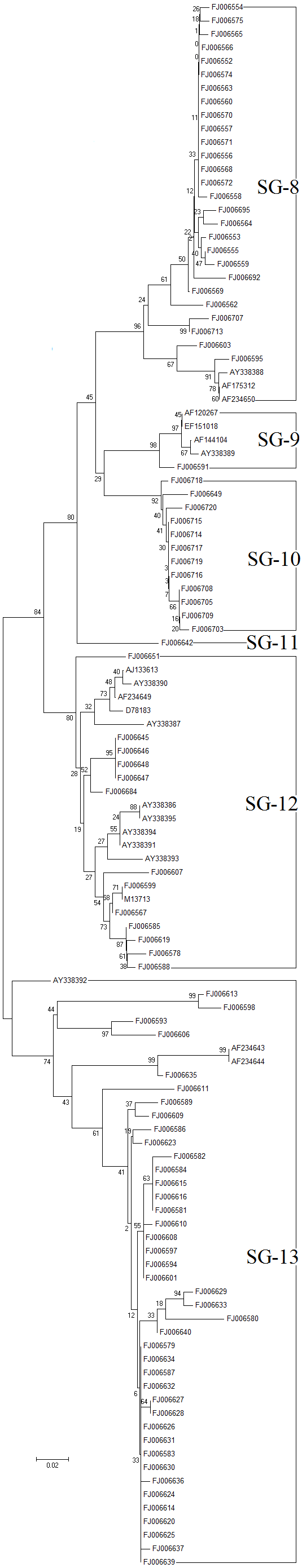

Supplement: Additional File 6 — Neighbour-joining tree of the γ-gliadins in pattern C8-P5, on the basis of alignment of the deduced mature protein sequences. SG-8 to SG-13 = subgroups described in Table 5. [file 1471-2164-10-168-S6.png]
