# Supplementary material for: The γ-gliadin multigene family in common wheat (Triticum aestivum) and its closely related species
Source: BMC Genomics. 2009 Apr 21;10:168. doi: 10.1186/1471-2164-10-168 (PMC2685405; doi:10.1186/1471-2164-10-168)
Supplement: Additional file 8 — The average proportions of eight essential amino acids. Avg-1, Avg-2 and Avg-3 indicate the average proportions of the eight essential amino acids in the 169 mature γ-gliadins (putatively functional), in the 18 γ-gliadins whose repetitive domains contain fewer than 85 amino acid residues (Their repetitive domains are included for data analyses; Table 6) and in the 169 mature γ-gliadins ignoring the repetitive domains, respectively. Data are mean ± SD (standard deviation). [file 1471-2164-10-168-S8.doc]

**Additional File 8 The average proportions of eight essential amino acids**

|  | Trp | Lys | Met | Thr | Phe | Val | Ile | Leu | Total |
| --- | --- | --- | --- | --- | --- | --- | --- | --- | --- |
| Avg-1 | 0.65±0.32 | 0.69±0.33 | 1.93±0.40 | 2.38±0.75 | 4.95±0.65 | 5.04±0.75 | 5.39±0.61 | 6.59±0.70 | 27.62±1.56 |
| Avg-2 | 0.89±0.47 | 1.09±0.42 | 2.21±0.33 | 2.04±0.67 | 3.65±0.79 | 6.18±0.64 | 6.65±0.40 | 7.92±0.73 | 30.63±1.08 |
| Avg-3 | 1.17±0.55 | 1.15±0.40 | 3.51±0.72 | 1.91±0.48 | 1.30±0.48 | 8.80±1.11 | 8.80±0.57 | 9.37±0.60 | 36.01±1.25 |

Avg-1, Avg-2 and Avg-3 indicate the average proportions of the eight essential amino acids in the 169 mature γ-gliadins (putatively functional), in the 18 γ-gliadins whose repetitive domains contain fewer than 85 amino acid residues (Table 6) and in the 169 mature γ-gliadins ignored the repetitive domains, respectively. Data are mean±SD (standard deviation).
