# Supplementary material for: The γ-gliadin multigene family in common wheat (Triticum aestivum) and its closely related species
Source: BMC Genomics. 2009 Apr 21;10:168. doi: 10.1186/1471-2164-10-168 (PMC2685405; doi:10.1186/1471-2164-10-168)
Supplement: Additional file 10 — Analogues of CD-toxic epitopes in γ-gliadins. The bold and italic letters indicate amino acid substitutions. [file 1471-2164-10-168-S10.doc]

**Additional File 10 Analogues of CD-toxic epitopes in γ-gliadins**

| Epitopes | FLQPQQPFPQQPQQPYPQQPQQPFPQ | LQPQQPFPQQPQQPYPQQPQ | FSQPQQQFPQPQ |
| --- | --- | --- | --- |
| Analogues | FLQP***R***QPFPQQPQQ***S***YPQQPQQPFPQ | LQP***R***QPFPQQPQQ***S***YPQQPQ | ***SK***QPQQ***P***FPQPQ |
|  | FLQP***R***QPFPQQPQQPYPQQPQQPFPQ | LQP***R***QPFPQQPQQPYPQQPQ | ***SQ***QPQQ***P***FPQPQ |
|  | F***I***QPQQPFPQQPQQ***T***YPQ***R***PQQPFPQ | ***P***QPQQ***T***FPQQPQ***L***P***F***PQQ***R***Q | ***FP***QPQQQFPQPQ |
|  | F***P***QPQQ***T***FPQQPQ***LSF***PQQPQQPFP***E*** | ***I***QPQQPFPQQPQQ***T***YPQ***R***PQ | F***P***QPQQQF***L***QPQ |
|  | F***P***QPQQ***T***FPQQPQ***L***P***F***PQQPQQPFPQ | ***Q***QPQ***L***PFPQQPQQP***F***PQ***PQ***Q | F***P***QPQQQ***L***PQPQ |
|  | FLQPQQPFPQQPQQPYPQQPQQ***L***FPQ | ***Q***Q***A***QQPFPQQPQQP***F***PQ***TQ***Q | F***P***QPQQQFPQPQ |
|  | FLQPQQPFPQQP***R***QPYPQQPQQPFPQ | ***P***QPQQ***T***FPQQPQ***LSF***PQQPQ | ***TQ***QPQQQFPQ***S***Q |
|  | F***P***QPQQ***T***FPQQPQ***L***P***F***PQQPQQPFPQ | ***P***QPQQ***T***FPQQPQ***L***P***F***PQQPQ | ***PQ***QPQQQFPQPQ |
|  |  | LQPQQPFPQQPQQPYPQ***E***PQ | F***P***QPQQQFPQPQ |
|  |  | LQPQQPFPQQPQQP***F***PQ***TQ***Q | ***SQ***QPQQQF***S***QPQ |
|  |  | LQPQQAFPQQPQQP***F***P***RTQ***Q | F***Q***QPQQ***P***FPQPQ |
|  |  | LQPQQ***A***FPQQPQQP***F***PQ***TQ***Q | ***PQ***QPQQ***R***FPQPQ |
|  |  | ***P***QPQQ***T***FPQQPQ***L***P***F***PQQPQ | ***LQ***QPQQ***PL***PQPQ |
|  |  | ***I***QPQQPFPQQPQQ***T***YPQ***R***PQ | ***PQ***QPQQ***P***FPQPQ |
|  |  | LQPQQPFPQQP***R***QPYPQQPQ | F***P***Q***T***QQQFPQPQ |
|  |  | ***P***QPQQ***T***FPQQPQ***L***PFPQQPQ | F***R***QPQQQ***L***PQPQ |
|  |  |  | F***P***Q***S***QQQFPQPQ |

The bold and italic letters indicate amino acid substitution
